# No Prognostic Impact of p53 and P-Glycoprotein Expression in Patients with Diffuse Large B-Cell Lymphoma

**DOI:** 10.5402/2011/670358

**Published:** 2011-12-22

**Authors:** Pairaya Rujirojindakul, Kumpol Aiempanakit, Kanita Kayasut, Arnuparp Lekhakula, Hutcha Sriplung

**Affiliations:** ^1^Department of Pathology, Faculty of Medicine, Prince of Songkla University, Hat Yai, Songkhla 90110, Thailand; ^2^Department of Internal Medicine, Faculty of Medicine, Prince of Songkla University, Songkhla 90110, Thailand; ^3^Epidemiology Unit, Faculty of Medicine, Prince of Songkla University, Songkhla 90110, Thailand

## Abstract

The aim of this study was to determine the clinical significances of p53 and p-glycoprotein (P-gp) expression on outcome predictors for patients with DLBC. We assessed the immunohistochemical expression of p53 and P-gp using formalin-fixed, paraffin-embedded specimens in 108 patients diagnosed with *de novo* DLBC. A high expression of p53 was found in 53.7% of the patients. No expression of P-gp was demonstrated in any of the specimens. There were no significant differences in the complete remission (CR) rate (*P* = 0.79), overall survival (OS) (*P* = 0.73), or disease-free survival (DFS) (*P* = 31) between the p53-positive and p53-negative groups. The final model from multivariate analysis that revealed poor performance status was significantly associated with CR (*P* < 0.001) and OS (*P* < 0.001). Moreover, the advanced stage was a significant predictor of DFS (*P* = 0.03). This study demonstrated no impact of the expression of p53 on either response or survival rates.

## 1. Introduction

Diffuse large B-cell lymphoma (DLBC) is the most common type of non-Hodgkin lymphoma (NHL) accounting for 50% of NHL in Thailand [1]. Although the outcomes of treatment were markedly improved by adding rituximab to standard CHOP regimen (R-CHOP) [2], the 5-year event-free survival rate was only 47% [3]. Stratifying newly diagnosed patients according to risk will provide invaluable information to achieve the optimal risk-adapted strategy. Since the International Prognostic Index (IPI) was introduced in 1993, the strong prognostic predictability has been proved [4, 5]. However, patients with high-risk IPI may be unsuitable for intensive therapy due to old age or poor performance status (PS). Therefore, additional prognostic factors reflecting tumor biology are needed to identify patients who might benefit from dose intensification or new targeted therapy.

The p53 tumor suppressor gene is located on the short arm of chromosome 17 (17p13.1). It plays a role in the regulation of cell survival acting as a cell-cycle checkpoint protein and apoptotic cell death. Therefore, the p53 protein contributes to preventing the replication of cells suffering from DNA damage. Loss of the p53 function may cause resistance to apoptosis that leads to treatment failure to DNA-damaging agents [6]. Thus, p53 inactivation may be associated with a poorer prognosis. However, it remains inconclusive whether the p53 expression is an independent outcome predictor in patients with non-Hodgkin lymphoma (NHL) [7].

In addition, another major reason for treatment failure is drug resistance. Several biological mechanisms are responsible for this problem. One of the most important reasons that has been extensively investigated is the multidrug resistance (MDR) gene. The classical MDR is related to the expression of the MDR-1 gene, which is located at chromosome 7 and encodes a 170-kDa membrane-associated P-glycoprotein (P-gp) [8]. The P-gp functions as an energy-dependent drug efflux pump and causes a reduction in intracellular accumulation of the drug. In NHL, varying incidences of P-gp expression were reported from 0 to 49% and its impacts on the response are controversial [9–11].

In this study, we evaluated p53 and P-gp expression, as well as clinical parameters in patients with DLBC. The purpose was therefore to verify their impacts on treatment outcomes.

## 2. Materials and Methods

From January 2003 to December 2006, 122 patients were enrolled at Songklanagarind Hospital, but only 108 patients had available tissue sections. The eligibility criteria were older than 18 years, newly diagnosed with DLBC, and had stage II–IV diseases. The patients with human immunodeficiency virus or primary extranodal lymphomas were excluded. For lymphoma immunophenotyping, monoclonal antibodies targeting CD3, CD5, CD20, and CD79a (Dako, Glostrup, Denmark) were used to determine the T- or B-cell lineage. This study was approved by the Ethics Committee of Prince of Songkla University.

Clinical stage was performed using the Ann Arbor staging system. All patients with stage II–IV were treated with a standard chemotherapy of cyclophosphamide, doxorubicin, vincristine, and prednisolone (CHOP) for at least six cycles. Rituximab was not routinely administered in Thailand. After completion of treatment, all patients were regularly followed up at intervals every few months for at least 5 years or until death.

### 2.1. Immunohistochemical Staining

Tumor samples were obtained by tissue biopsy at the time of initial diagnosis. Eighteen samples at the time of relapse for additional P-gp study were also included. The expressions of p53 and P-gp were analyzed by immunohistochemistry using the Envision technique. The immunohistochemistry was performed in formalin-fixed paraffin-embedded tissue sections. The 5-*μ*m-thick sections were cut on aminopropyltriethoxysilane-coated slides. They were deparaffinized in xylene and rehydrated through graded alcohol. Antigen retrieval was carried out by high-temperature and- pressure cooking in Tris EDTA pH 9 for 30 seconds. After rinsing in distilled water, the endogenous peroxidase activity was blocked by treating the slides with 3% hydrogen peroxide for 5 minutes. To inhibit nonspecific antibody binding, the tissue samples were reacted with nonimmune serum for half an hour in a moisture chamber. The sections were incubated overnight at 4°C with 1 : 250 and 1 : 40 dilutions of the monoclonal antibody of p53 (DO-7, DAKO, Glostrup, Denmark) and P-gp (Novocastra Laboratories, UK), respectively. After rinsing in phosphate-buffered saline solution, the sections were incubated with Envision for 30 min and then were rinsed in phosphate-buffered saline solution again. The color was developed using diaminobenzidine (DAB) solution for 5 min and counterstained with hematoxylin. Any brown nuclear and membrane staining of the tumor cells were taken as positive for p53 and P-gp, respectively.

### 2.2. Immunohistological Scoring

**Figure 1:**
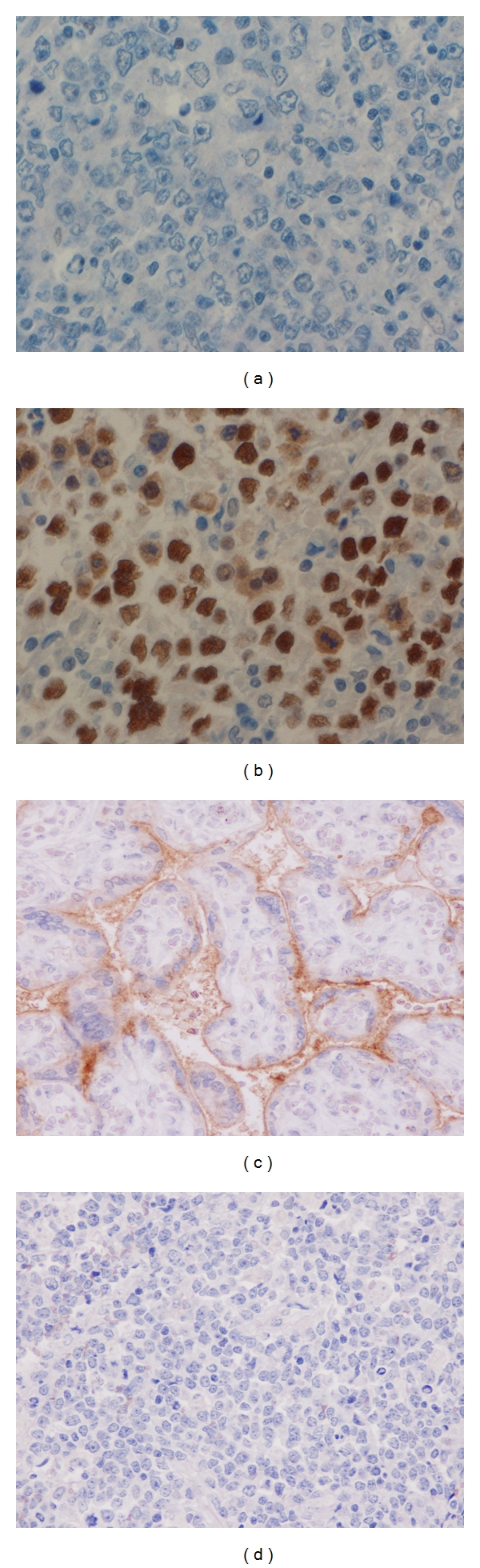
Immunohistochemical detection showing (×400) (a) negative for p53 staining in DLBC, (b) strongly positive for p53 staining in DLBC (+++), (c) placenta as a positive control for P-gp staining, (d) negative for P-gp staining in DLBC.

The scoring was analyzed in the area of highest protein expression scanning under low magnification (×100). Then, at least 500 cells under high-power field (×1000) were evaluated for a final score. Negative staining was made after carefully examining the entire section with high magnification. Expression of p53 and P-gp was semiquantitatively assessed without knowledge of the clinical outcome. The staining was graded on a scale of 1+, 2+, and 3+ when 10–24%, 25–49%, and 50% positive reactions were found, respectively [12]. Paraffin-embedded placenta was used as a positive control for P-gp (Figure 1(c)).

### 2.3. Statistical Analysis

Baseline categorical characteristics were compared among patients with and without overexpression of p53 using Chi-square. Logistic regression model was used to predict complete remission (CR). Univariate analysis of survival was performed with the Kaplan-Meier method. Overall survival (OS) was calculated as the time interval from the date of diagnosis to death or last follow-up. Disease-free survival (DFS) was defined as the time interval between CR and first progression/relapse or death from any cause. Kaplan-Meier methods were used to estimate time-to-event endpoints, including OS and DFS. Survival data between subgroups were compared using the log-rank test. Multivariate analyses for OS and DFS were performed using a Cox regression model with backward elimination. Critical *P*-values for entry and removal were 0.2 and 0.5, respectively. To test the main hypothesis, we forced the p53 into the model. Hazard ratios (HRs), 95% confidence intervals (95% CI) and *P*-values were obtained from the best-fit model. All the statistic analyses were calculated using the R program. A significance level of 0.05 was used throughout all statistical tests in the study.

## 3. Results

There were 122 DLBC patients diagnosed during the study period. However, only 108 patients with adequate paraffin-embedded tumor tissue sections were analyzed. To avoid bias, we compared the clinical characteristics and outcomes between the 108 and 14 patients and found no statistically significant differences (data not shown). There were 56 males and 52 females with a median age of 53.8 years (range 18–84 years). The majority of patients were treated with CHOP chemotherapy. Rituximab was administered in eight patients (7.4%). The median OS and DFS were 27.9 months (range 1–68 months) and 30.9 months (range 0–74 months), respectively with a median follow-up time of 19.7 months (range 0.3–66.2 months).

### 3.1. Expression of p53 and P-gp

Assessment of p53 expression was performed in 108 patients. Of these, 30 (27.8%) were p53− (Figure 1(a)), 5 (4.6%) were p53+, 15 (13.9%) were p53++ (Figure 1(b)), and 58 (53.7%) were p53+++ (Figure 1(a)). A comparison between clinical characteristics and p53 overexpression showed no significances in any of the variables (Table 1).

All tissue sections from patients with newly diagnosed and relapsed DLBC were negative for P-gp expression (Figure 1(d)). 

### 3.2. Univariate Analysis

Patients with bulky mass, poorer performance status (PS), and higher IPI were less likely to achieve CR. The results from univariate analysis showed that those who presented with at least one of the variables (more advanced stage, B symptoms, bulky mass, high LDH, poorer performance status, and/or higher IPI) were significantly associated with a shorter overall survival (OS). Simultaneously, only stage, B symptom, LDH, and IPI were significantly associated with the DFS (Table 2). There were no significant differences in the CR rate (*P* = 0.79), OS (*P* = 0.73), or DFS (*P* = 31) between the p53-positive (3+) and p53-negative groups (0–2+), even we tried to change the cut-off values to 1+ or 2+ (data not shown).

### 3.3. Multivariate Analysis

The preliminary regression model included the following variables: p53, sex, age group, stage, B symptoms, bulky mass, extranodal involvement, LDH and performance status. The final model revealed PS 2–4 was significantly associated with lower CR rate (OR 14.7, 95% CI 4.8–45.3, *P* < 0.001) and shorter OS (HR 5, 95% CI 2.8–9, *P* < 0.001). Moreover, the advanced stage was a significant predictor of DFS (*P* = 0.03). Patients with stage III had HR 3.1 (95% CI 1.3–7.5) while patients with stage IV had HR 2.7 (95% CI 1.1–7.1). 

## 4. Discussion

This study was undertaken to investigate the impact of p53 and P-gp expression as well as clinical parameters on treatment outcomes in patients with *de novo *DLBC. From multivariate analysis, our results indicate that only poor PS was independently associated with both CR and OS while the advanced stage was the independent predictor of DFS. Contrary to our expectations, the expression of p53 shows no impact on either response or survival rates. Furthermore, both initial and relapsed specimens were negative for P-gp expression.

Although there have been several studies on these markers, conflicting results have still existed. Moreover, independent associations between the individual markers and known clinical prognostic factors were not assessed. To address this, the relationship of the p53 expression with clinical endpoints was independently determined with clinical characteristics. We included only patients who were ethnic Thais and diagnosed with DLBC subtype to avoid confounding effects. In addition, they were homogeneously treated with the same anthracycline-containing regimen, and the treatment outcomes were analyzed based on complete follow-up data.

In our study, no expression of P-gp in any of the specimens was demonstrated. We tested three possible explanations to confirm the negative immunohistochemical results. First, we tried different concentrations of the P-gp antibody. Second, a few specimens with faint staining were repeated using an automatic immunostaining device (DAKO). These two hypotheses about the methods are unlikely to be responsible in the negative staining due to that the paraffin-embedded placenta showed positive staining. Another reasonable assumption is that the sample size was too small to detect the low positive expression rate of P-gp (95% confidence interval = 0–3.4% and 0–18.5% in the newly diagnosed and relapsed patients, respectively). Based on our knowledge, the negative result of P-gp from the immunohistochemical method has never been reported. One explanation may be from publication bias. Though the evidence shows that both RT-PCR and immunohistochemistry are reliable methods to detect MDR expression in lymphomas [10], the fresh specimens were not obtained to assess the expression of MDR-1 mRNA and P-gp that could strongly confirm the negative expression in this study.

Based on a cut-off value greater than 50% of positive immunostaining for p53, the positive rate of 53.7% is consistent with a report from Italy [13]. In addition, Chang et al. [14] demonstrated 58% of patients with high clinical stage DLBC stained positive for p53 using the cut-off value of 20% as a positive expression. Paik et al. [15] also showed overexpression of p53 in 56% of nodal DLBC with germinal centre phenotype based on nuclear staining more than 10%. However, our positive rate is higher than a population-based study using the cut-off value of 50% that shows that only 13% of 364 cases with B-cell lymphomas were positive [16]. Regarding the prognostic significance of p53, no significant relationship between p53 protein expression and any of the clinical variables and outcomes was confirmed in this study. These results were in concordance with a population-based study [16]. Similarly, nothing significant in survival was observed in previous studies [17, 18]. On the other hand, our results contradicted to the study that demonstrated the impact of p53 on survival [19, 20].

Altogether, this study provides evidence that both of the well-known biomarkers, p53 and P-gp, are not the independent predictors for treatment outcomes in these patients with DLBC. Nowadays, gene expression signatures provide evidences to predict the prognosis in patients with DLBC [21, 22]. Nevertheless, given the limitations of routine clinical utility, gene expression profiling in daily practice has allowed the immunohistochemistry to be of great practical value.

## Figures and Tables

**Table 1:** Clinical characteristics of the 108 patients according to p53 expression.

Variables	Immunostaining for p53 (no.)		*P*-value
	0–2+	3+	
Age group			0.69
≤60 years	20	47	
>60 years	10	31	
Sex			0.68
Male	17	39	
Female	13	39	
B symptoms			0.67
Absent	16	47	
Present	14	31	
Bulky mass (>6 cm)			0.39
Absent	25	57	
Present	5	21	
Extranodal involvement			0.86
Absent	11	29	
Present	19	49	
Serum LDH level			0.90
Normal	13	31	
High	17	47	
Ann Arbor staging system			0.12
II	11	37	
III	12	16	
IV	7	25	
ECOG performance status			0.46
0-1	25	58	
2–4	5	20	
International prognostic index			0.85
Low	8	15	
Low intermediate	15	28	
High intermediate	8	21	
High	5	14	

**Table 2:** Univariate analysis of CR, OS and DFS for 107 patients treated at Songklanagarind hospital.

Variables	Total	Univariate analysis		
		CR no. (%)	OS HR (95% CI)	DFS HR (95% CI)
Age group, y				
≤60		45 (68.2)	1.0	1.0
>60		26 (63.4)	1.4 (0.9–2.4)	1.2 (0.6–2.5)
Sex				
Male		39 (70.9)	1.0	1.0
Female		32 (61.5)	1.1 (0.6–1.8)	0.9 (0.4–1.8)
Stage				
II		35 (72.9)	1.0	1.0
III		18 (64.3)	2.0 (1.1–3.7)	3.2 (1.3–7.7)
IV		18 (58.1)	2.1 (1.1–3.9)*	2.5 (1.0–6.3)*
B symptoms				
Absent		45 (72.6)	1.0	1.0
Present		26 (57.8)	1.9 (1.1–3.1)*	2.6 (1.2–5.4)*
Bulky mass				
Absent		59 (72.0)	1.0	1.0
Present		12 (48.0)*	2.0 (1.1–3.6)*	0.5 (0.1–1.9)
Extranodal				
Absent		30 (75.0)	1.0	1.0
Present		41 (61.2)	1.5 (0.9–2.7)	1.4 (0.7–3.0)
LDH				
Normal		34 (77.3)	1.0	1.0
High		37 (58.7)	1.9 (1.1–3.3)*	2.4 (1.1–5.2)*
PS				
0-1		66 (79.5)	1.0	1.0
2–4		5 (20.8)**	4.8 (2.7–8.3)**	1.5 (0.4–4.8)
p53				
0–(2+)		36 (72)	1.0	1.0
3+		35 (61.4)	1.2 (0.7–2.0)	0.9 (0.4–1.9)
IPI				
Low		20 (87.0)	1.0	1.0
Low Int.		29 (78.4)	1.5 (0.6–3.4)	1.2 (0.4–3.6)
High Int.		16 (57.1)	3.6 (1.6–8.2)	3.3 (1.1–9.7)
High		6 (31.6)**	5.3 (2.2–12.6)**	3.4 (0.9–13.0)*

*P < .05, **P < .001.
